# In Silico Study of Correlation between Missense Variations of *F8* Gene and Inhibitor Formation in Severe Hemophilia A

**DOI:** 10.4274/tjh.galenos.2019.2019.0094

**Published:** 2020-05-06

**Authors:** Mostefa Fodil, Faouzia Zemani

**Affiliations:** 1 Higher School of Biological Sciences of Oran (ESSBO), Oran, Algeria; 2 Molecular and Cellular Genetics Laboratory, Oran University of Science and Technology - Mohamed Boudiaf (USTOMB), Oran, Algeria

**Keywords:** Hemophilia A, Missense variation, In silico analysis, Inhibitor formation, FVIII, Coagulation

## Abstract

**Objective:**

Deleterious substitutions of the *F8* gene are responsible for causing hemophilia A, which is an inherited bleeding disorder resulting from reduced or absent activity of the coagulant protein factor VIII (FVIII). The most important complication in treatment is inhibitor development toward therapeutic factor VIII. In this study, we aimed to analyze the effects of deleterious substitutions in the *F8* gene upon protein structure and function.

**Materials and Methods:**

All tests were conducted by computational methods from the CHAMP (CDC Hemophilia A Mutation Project) database. We performed an in silico analysis of deleterious variations using five software programs, Sift, PolyPhen-2, Align-GVGD, KD4v, and MutationTaster, in order to analyze the correlation between variation and the disease. We also studied the correlation between these variations and inhibitor formation.

**Results:**

Our analysis showed that these in silico tools are coherent and that there are more variations in the A than the C domains. Moreover, we noticed that there are more deleterious variations than neutral variations in each of the A and C domains. We also found that 13.51% of the patients suffered from a severe form of hemophilia A and that carriers of missense variations developed inhibitors. Also, for the first time, we determined that variation nature is not associated with inhibitor formation. Furthermore, this analysis showed that the risk of developing inhibitors increases when the variation causes a change of amino acid class.

**Conclusion:**

This study will help to correctly associate variations with inhibitor development and aid in early characterization of novel variants.

## Abstract (tr)

**Amaç:**

*F8* genindeki patolojik varyasyonlar, pıhtılaşma faktörü VIII’in (FVIII) azalmış ya da kaybolmuş aktivitesinden kaynaklanan ve kalıtsal bir kanama bozukluğu olan Hemofili A’ya neden olmaktadır. Tedavide en önemli zorluk, tedavi edici faktör VIII’e karşı inhibitör gelişimidir. Bu çalışmada *F8* gen varyasyonlarının protein yapısı ve fonksiyonu üzerine olan etkilerini incelemeyi amaçladık.

**Gereç ve Yöntemler:**

Tüm testler CHAMP (CDC Hemofili A Mutasyon Projesi) veri tabanından bilgisayar hesaplama yöntemleriyle yapıldı. Varyasyon ve hastalık arasındaki ilişkiyi araştırmak için beş farklı yazılım programı; Sift, PolyPhen-2, Align-GVGD, KD4v ve MutationTaster kullanarak, patojenik varyasyonların analizi yapıldı. İlave olarak bu varyasyonlar ve inhibitör oluşumu arasındaki ilişki de incelendi.

**Bulgular:**

Analizlerimiz bilgisayar tahmin araçlarının tutarlı olarak A bölgesinde, C bölgesine kıyasla daha fazla varyasyon olduğunu gösterdi. Ayrıca A ve C bölgelerinde nötral varyasyonlardan ziyade patojenik varyasyonlar bulunduğunu fark ettik. Ayrıca hastaların %13,51’inin ağır hemofili A olduğunu ve yanlış anlamlı varyasyon taşıyıcılarının inhibitör geliştirdiğini bulduk. Ayrıca ilk kez varyasyon türünün inhibitör oluşumu ile ilişkili olmadığını gösterdik. İlave olarak bu analiz, aminoasit değişimine yol açan varyasyonların inhibitör geliştirme riskini arttırdığını bize gösterdi.

**Sonuç:**

Bu çalışma inhibitör gelişimi ile varyasyonları doğru bir şekilde ilişkilendirmeye ve yeni varyasyonların erken karakterizasyonuna yardımcı olacaktır.

## Introduction

The X-linked bleeding disorder hemophilia A (HA) (OMIM #306700) is caused by a decrease or dysfunction in circulating blood coagulation factor VIII. This coagulation defect is present in 1/5000 of the male population [1,2]. According to the residual plasma FVIII coagulant activity (FVIII: C), HA can be classified into 3 forms: severe (FVIII: C<1%), moderate (1% <FVIII: C<5%), and mild (5% <FVIII: C<40%) [2]. Treatment of hemorrhages in hemophiliac patients consists of protein replacement therapy using plasma-derived or recombinant FVIII [3,4]. A serious complication of this therapy is the development of inhibitors (i.e. neutralizing alloantibodies against FVIII), which negate treatment benefits [2,5,6]. This process is observed in more than 30% of patients with severe HA. However, only 3% to 13% of patients with moderate and mild HA develop these inhibitors [7,8]. Several studies showed that determinants of inhibitor formation include environmental factors [9,10,11,12] as well as the patient’s genetic background. The type of variation in the *F8* gene is the strongest risk factor for inhibitor development [7,13]. A recent meta-analysis confirmed that the risk of patients with large deletions and nonsense variations was higher when compared with the risk of inhibitor development in patients with intron 22 inversion [13]. The same study showed that the risk of patients with intron 1 inversions and splice-site variations was equal, and the risk of patients with small deletions and insertions and missense variations was lower [13].

In our study, the role of *F8* missense variations in inhibitor risk was evaluated in a cohort of 407 patients with severe HA extracted from the  CDC Hemophilia A Mutation Project (CHAMP) database [14]. We have also assessed the impact of these missense variations on the structure and/or function of the FVIII protein using in silico programs.

## Materials and Methods

### Extraction of Variation Information

The variation information of *F8 *was retrieved from the CHAMP database for our analysis [14]. Among the 2537 variations listed in the CHAMP database until 2014, we selected 407 missense exon variations from severe hemophilia A patients for this study. Among these variants, 296 have known inhibitor status.

### Evaluation of the Variations

### Clustal W2

This software uses sequence homology to study the conservation between species during evolution [15]. The protein sequences of *Mus musculus, Rattus norvegicus, *and *Macaca fascicularis* were collected from the UniProt database (http://www.uniprot.org/) regarding their phylogenetic proximity. We then aligned these sequences to locate the variations relative to the important regions of the genome that are most conserved.

### SIFT

Sorting Intolerant From Tolerant  (SIFT) is a program based on sequence homology to predict whether an amino acid substitution will affect protein function [16]. The scores are classified as intolerant (0.00-0.05), potentially intolerant (0.051-0.10), borderline (0.101-0.20), or tolerant (0.201-1.00). A tolerant substitution does not have deleterious effects on protein function. On the other hand, intolerant substitution appears to have a partial or complete impact on the loss of protein function.

### PolyPhen-2

Polymorphism Phenotyping v2 (PolyPhen-2), available as software and via a Web server, predicts the possible impact of amino acid substitutions on the stability and function of human proteins using structural and comparative evolutionary considerations [17]. It is based on three types of information: the multiple alignment, structural information from the database structure (PDB), and the physicochemical properties of the amino acids. Predictions of a variation’s effect on protein structure are assigned as “probably damaging”, with a score of ≥2.000, and “possibly damaging”, with a score of 1.500-1.999, which means that these variations may affect protein function and/or structure. Finally, “benign”, with a score of 0.000-0.999, signifies no likely phenotypic effect.

### Align-GVGD

Align-GVGD is a freely available web-based program that combines multiple sequence alignment and biophysical characteristics of amino acids that are based on Grantham distance [18]. The Grantham distance calculates the physicochemical difference between two amino acids. If this distance is important, it means that the two amino acids are different. The results are established as classes C0 to C65. Classes C45 to C65 are more likely to affect the function, while classes C0 to C25 are less likely to affect the function.

### KD4v

KD4v is based on two complementary services: the first is similar to other prediction software such as SIFT and PolyPhen-2, while the second is based on the information and the three-dimensional (3D) structure to predict changes in size, charge, polarity, hydrophobicity, accessibility, and physicochemical properties of amino acids due to a missense variation [19]. KD4v predicts whether the variation is “neutral” or “deleterious” for the protein.

### MutationTaster

MutationTaster is a free web-based application to evaluate DNA sequence variants for their disease-causing potential. The software performs a battery of in silico tests to estimate the impact of the variant on the gene product/protein. This program was designed for the rapid assessment of the potential pathogenic alterations in DNA sequences [20]. It integrates information from different databases and biomedical analyses that include conservation during evolution, changes in splice sites, and the loss of protein function. MutationTaster predicts if the variation is “disease-causing” or just a “polymorphism”.

### Statistical Analysis

In order to evaluate statistical differences between different groups (presence or absence of inhibitors) we used the chi-square test (χ^2^). Classical chi-square evaluation is possible when numbers are greater than 5. An estimated p-value of less than or equal to 0.05 was considered to be statistically significant.

## Results

Among the 407 exon variations studied by ClustalW2, 378 (92.87%) are located in highly conserved regions. We applied five in silico tools, SIFT, PolyPhen-2, Align-GVGD, KD4v, and MutationTaster, to predict the effects of each variation on the protein function and/or structure (Table 1).

**Figure 1:**
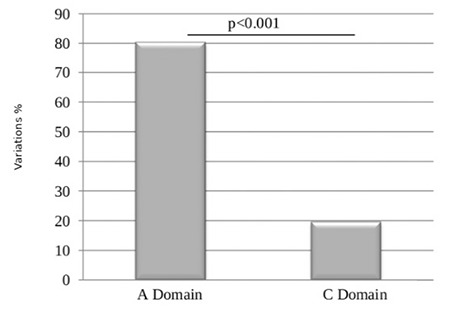
Variation distribution according to the A and C domains.

For the rest of the analysis, we chose to take into consideration the results of KD4V, since it is a software program based on structure homology and also considers the information on the three-dimensional structure. The results obtained allowed us to classify variations as deleterious or neutral. First, we studied the distribution of variations according to domains A (A1+A2+A3) and C (C1+C2) of the FVIII protein. The variations located on the B domain were not included in this study. In fact, the B domain does not play a major role in blood clotting. Our results showed that the A domain contains four times more variations than the C domain (Figure 1).

**Figure 2:**
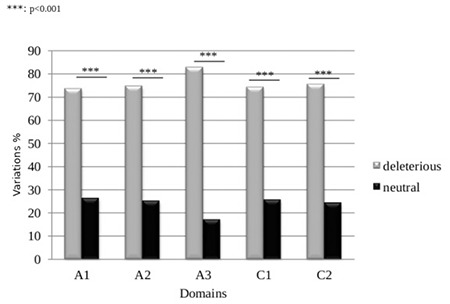
Deleterious and neutral variation distribution according to the A and C domains. ***: p<0.001.

We then examined the distribution of deleterious and neutral variations according to the A and C domains. Therefore, we calculated the frequencies of deleterious and neutral variations in each domain: A1, A2, A3, C1, and C2. We noticed that deleterious variations were significantly more prevalent than neutral variations in each domain (p<0.001) (Figure 2).

**Figure 3:**
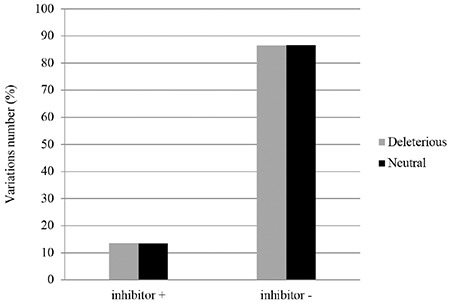
Correlation between variation character (deleterious/neutral) and inhibitor formation.

Among the 407 variations, 296 (72.73%) variants have known inhibitor status. Accordingly, 13.51% of patients developed inhibitors, while 86.49% did not (Table 2). In order to test the correlation between the nature of the variation (deleterious/neutral) and inhibitor formation, frequencies of deleterious and neutral variations in the two groups of patients were calculated (developing or not developing inhibitors). The results showed the absence of a correlation between inhibitor development and variation nature (Figure 3). In fact, in the group of patients developing inhibitors, there were no statistically differences between deleterious and neutral variations frequencies (13.54% vs. 13.43%; p=0.9). The same results were observed in the second group of patients that did not develop inhibitors (86.46% vs. 86.57%; p=0.9).

We then studied the impact of the localization of a variation on the A1, A2, A3, C1, and C2 domains on inhibitor formation. Therefore, for each domain we calculated the variant frequencies among the group of patients developing inhibitors. Frequencies of patients with missense variations located in the A3 and C2 domains were higher than those with variations located in the A1, A2, and C1 domains (Figure 3). However, this difference was not statistically significant (p=0.19).

Finally, we assigned four different classes of amino acids according to the properties of their side chains [class 1: hydrophobic (A, V, F, P, M, I, L, W); class 2: polar uncharged (S, Y, N, Q, C, T, H, G); class 3: acidic (D, E); class 4: basic (K, R)] Then we examined whether substitution caused changes in the amino acid class. The substitution of the wild-type amino acid by an amino acid from the same class gives an intra-class substitution. However, its replacement by an amino acid of another class results in inter-class substitution.

Comparison of all intra- and inter-amino acid substitutions showed that a significantly greater incidence of inhibitor formation was observed in the case of inter-amino acid substitutions than intra-amino acid substitutions: 28 (70%) vs. 12 (30%) (p=0.003, according to a normal distribution) (Table 3).

## Discussion

Alterations of the *F8* gene are extremely diverse. Many bioinformatics tools were used to assess the impact of these variations on protein function. These are based on the study of sequence conservation, amino acid physicochemical properties, and the information concerning the 3D structure of the FVIII protein [21,22,23]. In this perspective, we analyzed 407 variations extracted from the CHAMP database. We selected exon missense variations that are responsible for the severe form of HA.

The conservation analysis study focused on the most important regions that can influence the stability, the function, and the structure of the FVIII protein. The results obtained by ClustalW2 showed that 92.87% of the analyzed variations were located in highly conserved regions. Therefore, these variations are likely to have a very important deleterious effect on the function of the FVIII protein [24].

We then studied the distribution of the variations according to the A and C domains. The variations located in the B domain were not included in this study. In fact, the B domain does not play a major role in blood clotting, but it is involved in intra-cellular interactions such as the regulation of quality control and secretion. Therefore, it could be considered that missense variations located in the B domain can only affect the efficiency of secretion of FVIII [25,26]. Indeed, if a missense variation is identified in the B domain in a patient with HA, it would be necessary to look for other variations in the other domains of the *F8* gene.

Our results showed that there are four times more variations in the A domains than the C domains (80.34% vs. 19.66%, p<0.001). This is probably due to the fact that the peptide sequences of the A domains (1112 amino acids) are approximately three times longer than the peptide sequences of the C domains (312 amino acids) [27,28].

On the other hand, we studied the impact of the 407 variations using five in silico tools, SIFT, PolyPhen-2, Align-GVGD, KD4v, and MutationTaster, in order to predict deleterious and/or damaging effect of variations. The combination of the results obtained by these software programs showed that there were more deleterious than neutral variations. This observation was in keeping with the patients’ phenotypes as they developed the severe form of HA. According to KD4v results, we noticed that there were three times more deleterious variations than neutral variations. This difference was still valid for each of the A domains (A1, A2, and A3) and C domains (C1 and C2) (p<0.001). This observation was in correlation with the results obtained by MutationTaster, which predicted that 87.22% of the variations were disease-causing and 12.78% were polymorphisms. Regarding the neutral variations, they probably represent polymorphisms that are not responsible for the disease. In fact, it has been reported that in 2% to 18% of patients with HA, no genetic alterations were detected except polymorphisms [29,30,31]. Moreover, the A and C domains have important interaction sites. Indeed, the activation sites of the FVIII protein by thrombin are located in the A domain (Arg372, Arg740, and Arg1689) [32]. Consequently, if a variation affects one of these sites, the FVIII will not be activated and the tenase complex will not activate the FX. This induces the arrest of the coagulation cascade [33]. Furthermore, the C domain interacts with von Willebrand factor and the phospholipid membrane. These interactions are responsible for maintaining the stability and structure of the FVIII protein [7]. Besides, the A domain has six disulfide bonds (Cys-Cys) and the C domain contains only two. Those bridges are responsible for the protein stability and risk being broken because of missense variations [34].

Furthermore, in order to study the correlation between the impact of the variations and inhibitor formation, we examined the 296 variations that have known inhibitor status. We have shown that 13.51% of the patients with a severe form of HA carrying missense variations developed inhibitors. This frequency is higher than that found in Oldenburg and Pavlova’s study, where HA patients with missense variations had a risk of 5% of developing inhibitors [7]. This difference can be explained by the fact that our study concerns only the severe form of HA. In a recent study, Spena et al. [36] evaluated the association between *F8* gene variants and inhibitor development by analyzing 231 causative variants, grouped as low-risk and high-risk variations according to Gouw et al. [35]. Only a small difference was observed in the cumulative inhibitor incidence [32.0% (95% CI=18.9 to 45.1) vs. 37.9% (95% CI=29.9 to 45.9)] for low- and high-risk variations classified corresponding to a hazard ratio of 1.35 (95% CI=0.78-2.35) [36].

Otherwise, according to the hypothesis of Schwaab et al. [37], the low risk of developing inhibitors in patients with missense variations is due to the fact that patients with missense variations synthesize some endogenous protein that, although functionally altered, are sufficient to induce immune tolerance.

We supposed that a deleterious variation that alters the protein function and structure might increase the risk of developing inhibitors. We noticed that there were no correlations between deleterious missense variations and inhibitor formation (p=0.9).

We then studied the association between the location of a variation in the A and C domains with inhibitor formation. We observed that these variations are located in different domains. There were more variations located in the A3 and C2 domains (respectively 20.55% and 18.75%) than the other domains. However, this difference was not significant (p=0.19). Indeed, the FVIII inhibitors recognize epitopes on all the domains [38].

Finally, we analyzed the impact of a change of physicochemical properties of amino acids due to missense variations according to inhibitor formation. Our data showed that the risk of developing inhibitors increases when the variation causes a change of amino acid class (70% vs. 30%; p=0.003). These results support those of Schwaab et al. [37] study (91.5%). This percentage decreases (8.5%) in the case of patients with missense variations that do not cause changes in amino acid class [37].

## Conclusion

Our study showed that there are more variations in the A than the C domain. Moreover, we noticed that there are more deleterious than neutral variations in each of the A and C domains. For the first time, we have determined that variation nature is not associated with inhibitor formation. This study showed that variations in patients developing inhibitors are localized on both A and C domains of FVIII. Finally, we showed that the risk of developing inhibitors increases when the variation causes a change of amino acid class.

This analysis showed that combining information from different tools may facilitate a better understanding for predictive accuracy in determining the functional impact of a given variation.

## Figures and Tables

**Table 1:**
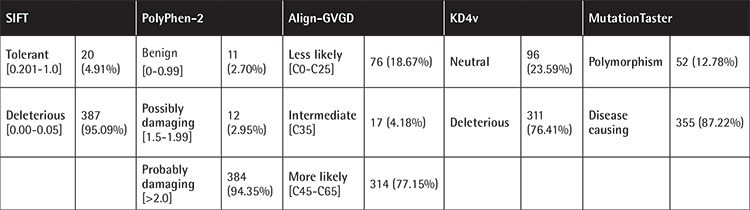
Prediction results of studied variations’ effects on the protein function and/or structure.

**Table 2:**
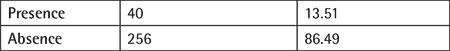
Distribution of single-nucleotide polymorphisms in patients with or without inhibitors.

**Table 3:**
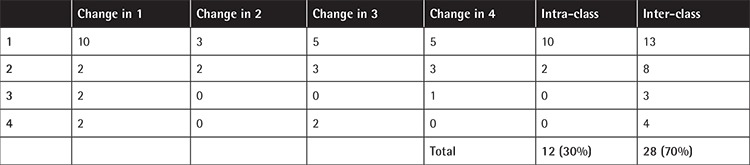
Variation distribution in the patients that developed inhibitors according to the change of amino acidic class. 1: Hydrophobic (A, V, L, I, M, P, F). 2: Polar uncharged (S, Y, N, Q, C, T, H, G). 3: Acidic (D, E). 4: Basic (K, R).

**Figure 4:**
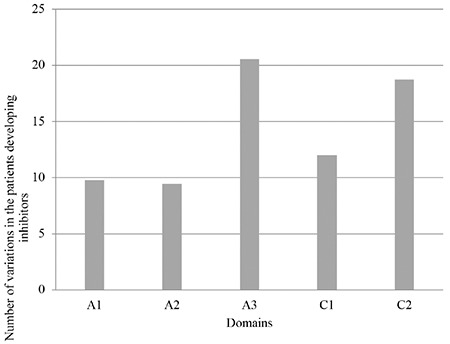
Correlation of variation localization on each domain with inhibitor formation.
